# Severe deterioration of left ventricular function after left bundle branch area pacing: A rare case report

**DOI:** 10.1016/j.hrcr.2025.10.036

**Published:** 2025-10-31

**Authors:** Thomas Koh, Sachin Nayyar

**Affiliations:** 1 Department of Cardiology, Gold Coast University Hospital, Southport, Queensland, Australia; 2 School of Medicine and Dentistry, Griffith University, Gold Coast, Queensland, Australia

**Keywords:** Left bundle branch area pacing, Pacemaker-induced cardiomyopathy, Left ventricular dysfunction, Case report, Conduction system pacing

**Key Teaching Points**

• Although left bundle branch area pacing (LBBAP) is a physiological pacing strategy, cardiomyopathy progression may still happen in select patients. Randomized controlled trials are needed to better evaluate its long-term safety and efficacy.
• Patient-specific factors such as preexisting myocardial scar, high ventricular pacing burden, and progression of primary cardiomyopathy may negatively affect outcomes after LBBAP.
• Closer monitoring of left ventricular function may be necessary in patients with risk factors of cardiomyopathy even after LBBAP.

## Introduction

Left bundle branch area pacing (LBBAP) has emerged as a promising alternative to conventional right ventricular pacing (RVP) and biventricular pacing for treating patients with narrow QRS complexes.^1^ LBBAP provides physiological pacing by depolarizing ventricles via the Purkinje system, preserving cardiac electrical and mechanical synchrony, which is often disrupted with conventional pacing methods, resulting in pacemaker-induced cardiomyopathy (PICM).^1^ Several large observational studies have shown superior outcomes in reducing heart failure–related hospitalizations and all-cause mortality for patients undergoing LBBAP vs RVP.^2^^,^^3^ Despite these undisputed benefits, the efficacy of LBBAP in preventing cardiomyopathy progression remains uncertain given its novelty. In this case report, we present a unique case of severe deterioration of left ventricular (LV) function observed after LBBAP implantation. This case contradicts the assumption of universal benefits surrounding LBBAP and highlights areas of future research.

## Case report

A 79-year-old man presented with symptomatic chronic slow atrial fibrillation, intermittent complete heart block with periods of profound bradycardia, and multifocal premature ventricular contraction (PVC) burden of 13% per 24 hours, necessitating permanent pacemaker (PPM) implantation. His medical history was significant for ischemic heart disease, systemic hypertension, and stage 3B chronic kidney disease.

**Figure 1:**
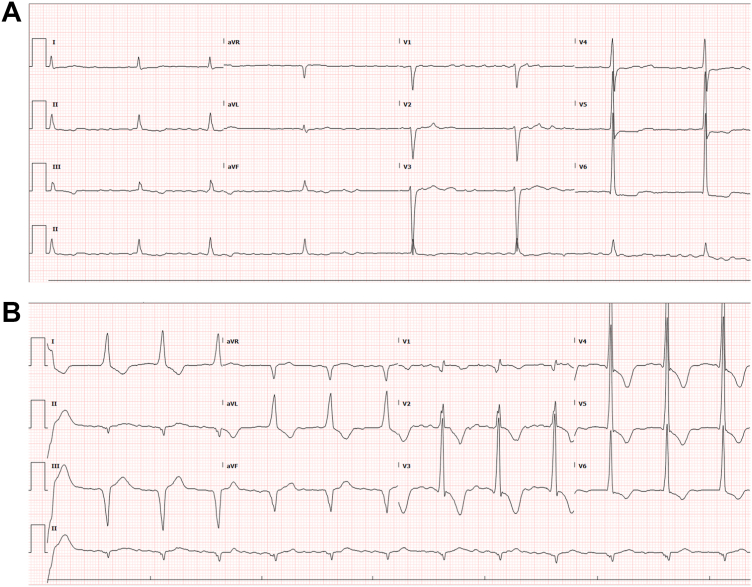
**A:** Baseline 12-lead electrocardiogram showing atrial fibrillation at 51 beats per minute and QRS duration of 108 ms. **B:** 12-lead electrocardiogram after permanent pacemaker insertion showing paced-QRS duration of 125 ms.

Preprocedural evaluation revealed an electrocardiogram (ECG) showing atrial fibrillation at a ventricular rate of 51 beats per minute and QRS duration (QRSd) of 108 ms (Figure 1A). Given these findings, a single-chamber LBBAP procedure was performed (Biotronik Evity SR-T). The lead (Biotronik Solia S60) was successfully screwed into the ventricular septum, achieving left bundle (LB) capture with a threshold of 0.5 V at 0.4 ms, impedance of 331 ohms, R-wave amplitude of 14.7 mV, and a paced QRSd of 125 ms (Figure 1B).

**Figure 2:**
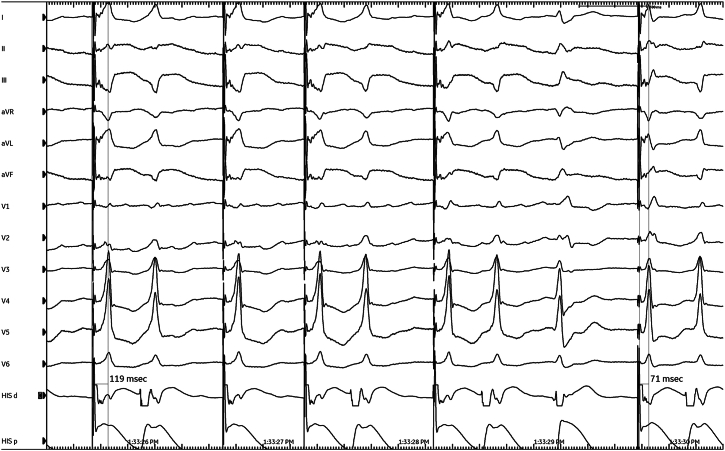
Implant procedure record showing abrupt reduction in left ventricular activation time in lead V6 and fixation beats during ventricular-lead advancement into the septum.

**Figure 3:**
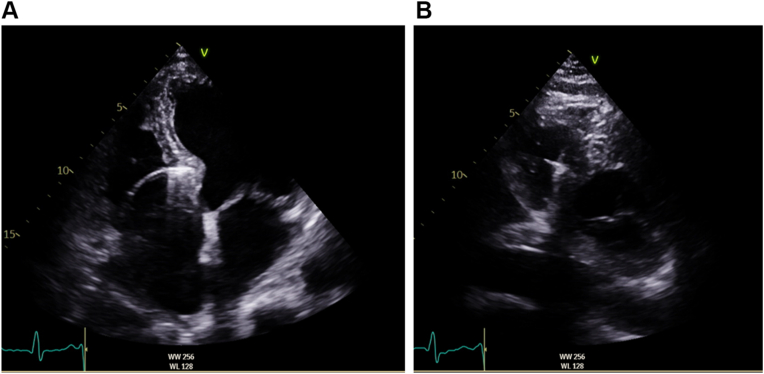
**A:** Apical 4-chamber view showing the placement of the ventricular lead in the left subendocardial septum immediately after implant. **B:** Short-axis view showing the placement of the ventricular lead in the left subendocardial septum immediately after implant.

The LB branch (LBB) capture was confirmed by unipolar pacing, demonstrating an abrupt reduction in LV activation time (LVAT) in lead V6 from 119 ms to 71 ms during progressive ventricular-lead advancement into the septum (Figure 2). Postprocedure echocardiography also confirmed appropriate positioning of the LB lead tip within the LV subendocardium with LV ejection fraction (LVEF) of 40%–45% (Figure 3A and 3B, Supplemental Video 1). Echocardiography performed 3 years ago also showed an LVEF of 45%, showcasing stability in LV function before device implantation. The patient was only on antihypertensive therapy consisting of irbesartan 300 mg, hydrochlorothiazide 25 mg, and amlodipine 5 mg.

**Figure 4:**
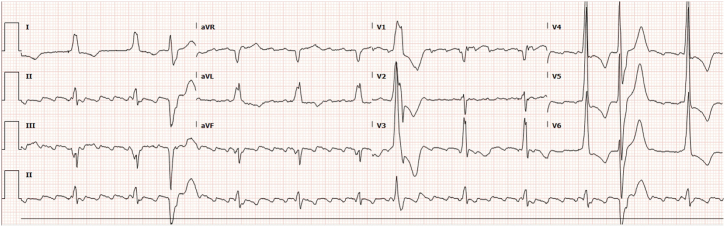
12-lead electrocardiogram at 8 months after implant showing a stable paced-QRS duration of 125 ms and a short left ventricular activation time in lead V6.

8 months after the PPM implant, the patient presented with worsening exercise tolerance, new orthopnea, and New York Heart Association class IV dyspnea. Physical examination findings showed jugular venous pressure elevation to the jaw, bilateral lung crackles, and significant peripheral edema, consistent with decompensated heart failure. ECGs showed a persistently stable paced-QRS complex with a maximum duration of 125 ms (Figure 4) and multifocal PVCs. The LVAT in lead V6 on the surface ECG also remained unchanged, indicating stable lead position and LB capture. Owing to the use of a single-chamber device, PVC burden could not be accurately assessed, but there were no nonsustained or sustained high ventricular rates. All device parameters had remained stable with multiple device interrogations over 8 months.

**Figure 5:**
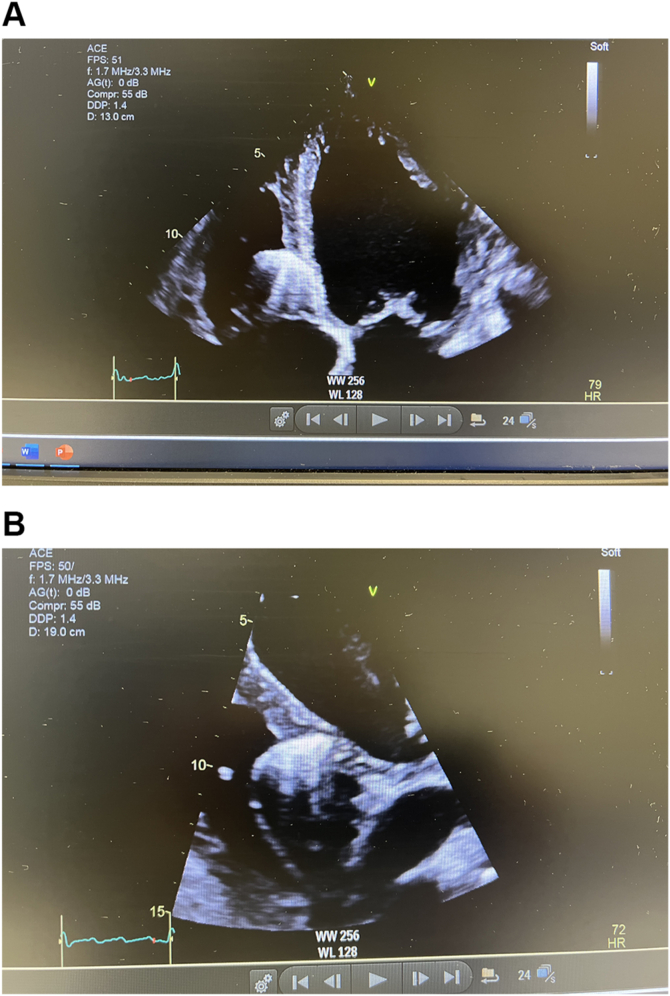
**A:** Apical 4-chamber view 8 months after permanent pacemaker implant showing stable ventricular-lead position in the left subendocardium. **B:** Modified parasternal view 8 months after implant showing a stable ventricular-lead position in the left subendocardium.

Echocardiography at this time demonstrated severely impaired LV function with an LVEF of 12% with global LV hypokinesia. Moderate aortic regurgitation was noted but unchanged from previous imaging. The lead tip remained in the LV subendocardium, consistent with the immediate postimplant position (Figure 5A and 5B and Supplemental Video 2). His medications were escalated to heart failure guideline-directed medical therapy, including angiotensin receptor–neprilysin inhibitor 24/26 mg, spironolactone 25 mg, bisoprolol 5 mg, and dapagliflozin 10 mg, with good symptom response. Coronary angiography was not considered owing to the global nature of ventricular dysfunction and lack of signs of acute coronary syndrome. Cardiac magnetic resonance imaging was planned but could not be completed at the time of writing this report because there were strict institutional restrictions on cardiac magnetic resonance imaging in the presence of an intracardiac device in a pacing-dependent patient. Furthermore, after shared decision making with the patient, cardiac resynchronization upgrade with additional coronary sinus lead (LBB-optimized cardiac resynchronization therapy [LOT-CRT]) was not pursued because paced QRSd was 125 ms with adequately short LVAT of 71 ms. Any further LV resynchronization and QRSd shortening were of doubtful consequence.

## Discussion

Approximately 40% patients fail to respond or deteriorate after conventional biventricular pacing,^2^ despite a well-positioned coronary venous lead, perhaps owing to residual dyssynchrony and extensive scarring.^4^ In this article, we reported a rare case of rapidly deteriorating LVEF under the watch of optimal LBBAP, with LVEF decreasing from 45% to 12% over 8 months despite a previously stable LVEF. The current literature does not provide a clear pathophysiological mechanism for LBBAP-induced PICM, and the observed decline may instead represent progression of the underlying cardiomyopathy. Nevertheless, this case is thought provoking that LBBAP may not be universally preventative.

### Shared risk factors

RVP-induced PICM has been well-documented in numerous studies, primarily caused by LV dyssynchrony, which leads to adverse cardiac remodeling.^1^^,^^5^ Risk factors for RVP-induced PICM include high pacing burden, wide baseline QRSd, preexisting atrial fibrillation, frequent PVCs, and impaired LVEF before PPM implantation.^3^ Given that attributing our patient’s LVEF decline directly to LBBAP-induced PICM is controversial, the temporal relationship of LBBAP and the new rapid decline in LVEF after implantation raises this possibility. In addition, the high pacing burden, baseline decreased LVEF, and preexisting atrial fibrillation may have increased the risk of LBBAP-induced PICM.^5^ PVCs have long been implicated in reversible LV dysfunction. However, multifocal PVCs may be a marker of underlying myocardial disease rather than a sole driver of dysfunction.

### Proximal vs distal conduction system capture

There exists only 1 case report of LVEF deterioration after LBBAP implantation. Meyers and Liu^6^ reported a case of probable LBBAP-induced PICM, where the ventricular-lead placement was too far from the tricuspid annulus, resulting in a QRS width of 135 ms and subsequent cardiac dyssynchrony. In contrast, our patient’s post-PPM QRSd of 125 ms and the lead positioned near the tricuspid annulus, as evidenced by echocardiography, suggest a lower likelihood of dyssynchrony-induced cardiomyopathy.

### Deep septal vs true LBBAP

When discussing LBBAP outcomes, the distinction between deep septal and true conduction system pacing is crucial. Chen et al’s^7^ observational study demonstrated that deep septal pacing was associated with worse outcomes than LBBAP or LV septal pacing. This may be attributed to subtle electromechanical dyssynchrony caused by incomplete conduction system capture that may not be apparent on surface ECG, leading to mechanical discoordination. In our case, the implant ECG record confirmed true conduction system capture based on established criteria, which had remained durable on follow-up suggesting that the patient received satisfactory pacing strategy to mitigate ventricular dyssynchrony. Despite this, the patient’s condition deteriorated, raising questions about other hidden factors influencing LBBAP outcomes.

### Residual dyssynchrony

Obligatory LBBAP from the left posterior fascicle trunk may inherently introduce dyssynchrony owing to delayed septal and left anterior fascicular conduction.^8^ This was evident in our patient’s initial postimplant ECG as septal and left anterior fascicular block (Figure 1B). However, given the absence of intraventricular conduction disease at baseline (Figure 1A) and resolution of septal and fascicular blocks on follow-up ECGs (Figure 4), it becomes difficult to attribute the LVEF decline to fascicular dyssynchrony, although its intermittent form cannot be entirely excluded without continuous 12-lead ECG monitoring. Anodal capture of the RV septum during bipolar LBBAP can also compromise LV pressure improvement after LBBAP owing to competing RV to LV wavefront across the interventricular septum. However, its contribution toward chronic PICM needs further studies.^9^

### Myocardial scar

Furthermore, underlying myocardial substrate abnormalities such as septal scar burden may have contributed to our patient’s poor response to LBBAP. Scar tissue within the interventricular septum can impair physiological conduction and diminish the benefits of LBBAP. Chen et al^10^ demonstrated that lower septal scar burden predicted better echocardiographic response to LBBAP. Nonetheless, the establishment of a new septal scar is less likely in our patient, given that severe ventricular dysfunction was observed precipitously within a few months after implant and there was no concurrent widening of the paced-QRS morphology on follow-up.

### Optimization of QRS Response after LBBAP

The data remain limited regarding crossover options for LBBAP to LOT-CRT (additional coronary sinus lead) during follow-up.^11^ There are no randomized studies in this area; most of the recommendations are based on retrospective analyses of patients who during follow-up were crossed over to a different anatomic pacing approach that proved feasible and/or subsequently successful. Consensus documents propose adding LV coronary vein pacing to LBBAP when there are technical difficulties at acute implant and the electrical criteria of LBBAP are suboptimal. An empirical upfront LOT-CRT approach is also expected to achieve meaningful LV dP/dtmax improvement and QRS shortening in patients with baseline wide QRS of >171 ms.^9^ However, additional synchronization is not expected when true LBB capture has been achieved with a narrow QRS of <130 ms, which has subsequently remained durable. In our patient, despite clinical deterioration, the paced QRSd remained at 125 ms with short LVAT confirming durable LBB capture. On this basis, after shared decision making, we judged that LOT-CRT is unlikely to improve clinical outcomes in this case.

## Conclusion

This case report is a rare example of cardiomyopathy after LBBAP. As a single observational case, it does not establish a causal relationship between LBBAP and cardiomyopathy progression. The observed LVEF decline may have been a result of a multifactorial progression of preexisting disease that cannot be definitively confirmed; however, the temporal association between optimal LBBAP implantation and rapid functional decline is intriguing. This case underscores that even “optimal” conduction system pacing may fail to be protective in patients with complex myocardial substrates.

## Disclosures

The authors have no conflicts of interest to disclose.
