# Supplementary material for: Severe deterioration of left ventricular function after left bundle branch area pacing: A rare case report
Source: HeartRhythm Case Rep. 2025 Oct 31;12(1):87–91. doi: 10.1016/j.hrcr.2025.10.036 (PMC12925730; doi:10.1016/j.hrcr.2025.10.036)
Supplement: Supplementary Video Legends [file mmc3.docx]

Supplementary file

**Legend**

Video 1: Echocardiogram immediately post-procedure showing LVEF ~45% and appropriate positioning of ventricular lead in the left subendocardial septum.

Video 2: Echocardiogram at 8 months after implantation showing lead tip in LV subendocardium
